# Increased Duration of Total Hip and Total Knee Arthroplasty Surgery Increases the Risk of Post-Operative Urinary Retention: A Retrospective Cohort Study

**DOI:** 10.3390/jcm13113102

**Published:** 2024-05-25

**Authors:** Edwin Yuen Hao Tong, Mariam Sattar, Iain A. Rankin, George Patrick Ashcroft

**Affiliations:** Trauma and Orthopaedics, Aberdeen Royal Infirmary, Aberdeen AB25 2ZN, UK; mariam.sattar@nhs.scot (M.S.); george.ashcroft@nhs.scot (G.P.A.)

**Keywords:** arthroplasty, urinary retention, orthopaedics

## Abstract

**Background:** Post-operative urinary retention (POUR) is a common complication following total hip arthroplasty (THA) and total knee arthroplasty (TKA). Spinal anaesthetic has been associated with an increased risk of POUR, whilst other risk factors remain unclear. This study aims to identify modifiable and non-modifiable risk factors of POUR for THA and TKA patients. **Methods:** A single-centre retrospective cohort study of patients admitted to our hospital over the course of 6 weeks from September to October 2021 for a THA or TKA. All patients who underwent elective THA/TKA were included, and trauma cases were excluded. **Results:** Ninety-two consecutive patients were included in this study. The overall rate of POUR was 17%. A shorter operative duration resulted in a reduced risk of POUR (median duration of non-retention patients, 88 min vs. 100 min POUR patients; odds ratio, 0.97; 95% CI, 0.95–0.99, *p* = 0.018). The median bladder volume of patients with urinary retention at the point of diagnosis was 614 mL (range, 298–999 mL). The arthroplasty type, anaesthetic technique, pre-operative morphine use, body mass index, age, cardiovascular disease, and renal disease were found to have no significant association with POUR. **Conclusions:** A reduced operative time of arthroplasty surgery is associated with a decreased risk of POUR. Patients with a prolonged operative time should have an increased frequency of micturition monitoring in the immediate post-operative period.

## 1. Introduction

The gold standard for the treatment of end-stage knee or hip disease would be to perform a total joint replacement (TJR) [1,2,3,4]. However, post-operative urinary retention (POUR) commonly occurs in patients undergoing TJR, with an incidence of 0 to 75% [5]. Studies have also shown that both total knee arthroplasty (TKA) and total hip arthroplasty (THA) may result in POUR [5,6].

An average bladder capacity ranges between 400 and 600 mL, with an indication to void at approximately 150 mL and the feeling of fullness at 300 mL [7]. A multitude of factors are believed to contribute to the development of POUR, including a history of urological conditions, increased fluid requirements perioperatively, and the combined use of analgesics, opiates, and anaesthesia [8]. These factors may result in bladder overdistension, a reduced awareness of bladder filling, reduced bladder contractility, and a micturition reflex [7]. Moreover, the delayed diagnosis of POUR may result in an acontractile bladder and the long-term impairment of detrusor muscles [9]. When recognized, POUR is managed by performing urinary catheterization. However, the definitive volume of urine detected in a bladder indicative of catheterization is unknown, resulting in the derivation of different criteria for catheterization [8]. Urinary catheterization is also associated with an increased risk of urinary tract infections (UTIs) [10], which can predispose patients to haematogenous bacteraemia [11] and subsequent joint injection [12].

The conventional methods of anaesthesia administered to patients undergoing total hip arthroplasty (THA) or total knee arthroplasty (TKA) typically involve either general anaesthesia (GA) or spinal anaesthesia (SA). Factors influencing the choice of anaesthesia include ease of the post-operative recovery, occurrence of post-operative urinary retention (POUR), nausea, and vomiting [13]. Generally, GA is more frequently favoured due to its relatively quicker onset of action. However, for THA and TKA procedures, SA has demonstrated efficacy and reliability, boasting a success rate exceeding 90% and superior control over post-operative nausea and vomiting. Additionally, a thorough examination of randomized controlled trials (RCTs) has indicated that SA contributes positively to reducing the risk of acute kidney injury (AKI), although no significant disparities were observed in the intraoperative hypotension risk when compared to GA [14]. Moreover, morphine is often administered into the subarachnoid space to augment the analgesic effects of SA, thereby facilitating better pain management. Nevertheless, prior studies have shown that combining SA with intrathecal morphine leads to a higher incidence of urinary retention, necessitating urinary catheterization [15].

Some other possible risk factors for the development of POUR include an increasing age, male gender, and a history of bladder outflow problems or surgeries [16]. Therefore, it would be helpful for orthopaedic surgeons to be able to examine and identify patients with perioperative risk factors and take preventive measures to reduce the occurrence of POUR.

## 2. Method

### 2.1. Study Population

This is a single-centre retrospective cohort study of all patients admitted to Woodend General Hospital, Aberdeen, United Kingdom, over a period of 6 weeks from 16 September 2021 to 29 October 2021 for either THA or TKA. The inclusion criteria comprised patients who were electively scheduled for a total hip or total knee arthroplasty, while cases involving trauma and paediatrics were excluded as they were not performed within this hospital. We obtained confirmation from the local ethics committee stating that patient consent was not required, as patient data were collected anonymously by accessing patient records. Additionally, ethical approval was deemed unnecessary due to the retrospective audit nature of the project’s design.

### 2.2. Outcomes

We identified 3 distinct outcomes for use in our study. Our primary outcome variable was the prevalence of POUR. Additionally, our secondary outcome was the bladder volume of patients prior to catheterization for POUR. For the subgroup of patients with POUR, we also considered if they experienced complications, such as acute kidney injury (AKI) and urinary tract infection (UTI).

### 2.3. Data Collection

The patient demographic and operation details were obtained from electronic health records (TrakCare^®^, Opera). We also reviewed patients’ paper notes, as certain data were not electronically updated at the time of data collection. Collected data included whether catheterization was performed pre- or post-operatively, whether a catheter was required, or a history of an indwelling catheter. Other noted variables included the timing and duration of catheterization, duration of surgery, type of surgery, bladder scan results, age, sex, body mass index (BMI), urinalysis, UTI, joint sepsis, anaesthetic type, pre-operative morphine requirement, past medical and urological history—particularly benign prostatic hyperplasia (BPH), urinary incontinence, and urinary retention.

### 2.4. Statistical Analysis

Categorical variables were analysed using chi-square testing and presented as counts and percentages. Continuous variables were assessed for normality and were analysed using a *t*-test and are presented as the mean with the SD. Univariate logistic regression was used to determine the effects of gender, type of arthroplasty, use of pre-operative morphine, age, BMI, duration of surgery, and length of hospital stay on the occurrence of POUR. Any two-sided *p*-values less than 0.05 were considered significant. All analyses were conducted using Statistical Package for Social Science (SPSS) version 20.0 software (IBM, Armonk, NY, USA).

## 3. Results

A total of 92 patients were included in this study. Among them, 16 (17.4%) experienced POUR and subsequently underwent catheterization. The mean patient age was 69.9 years (69.9 ± 10.9 years), and the mean BMI was 30.9 kg/m^2^ (30.9 ± 5.5 kg/m^2^). On average, patients were admitted for 3.3 days (3.3 ± 4.5 days). Risk factors contributing to post-operative urinary retention (POUR) were categorized into modifiable and non-modifiable factors. Non-modifiable risk factors included fundamental demographics, such as gender, age, BMI, comorbidities, type of arthroplasty, and duration of surgery. Modifiable risk factors encompassed the type of anaesthesia administered.

### 3.1. Non-Modifiable Risk Factors

There was no statistically significant difference in the development of POUR between male and female patients (*p* = 0.73). Additionally, neither age (*p* = 0.18), BMI (*p* = 0.72), nor the type of arthroplasty (*p* = 0.31) significantly varied between patients who developed POUR and those who did not (Table 1).

The incidence of POUR was lower in patients with a previous history of CVD, with 12 cases compared with 45 cases in those who did not develop urinary retention post-operatively. Furthermore, our data showed a lower incidence of CKD in the cohort who developed retention compared to those who did not, with 1 patient and 11 patients, respectively (*p* = 0.3).

The most significant non-modifiable risk factor for developing POUR was the duration of surgery (105 ± 34 vs. 89 ± 19, [*p* = 0.012]). As this was the most significant finding regarding risk factors, we conducted an exploratory univariate logistic regression. This confirmed that a shorter operative duration results in a lower risk of POUR (OR = 0.97, CI = 0.95–1.00, [*p* = 0.018]) (Table 2).

### 3.2. Modifiable Risk Factors

Our data showed no statistically significant difference in the incidence of POUR when comparing patients who had SA or GA (*p* = 0.17). Additionally, patients who were administered pre-operative morphine did not have a higher incidence of POUR (*p* = 0.88). The length of hospital stay was also not significantly increased in the cohort of patients who developed POUR (*p* = 0.51)

### 3.3. Bladder Volume Prior to Catheterization

**Figure 1:**
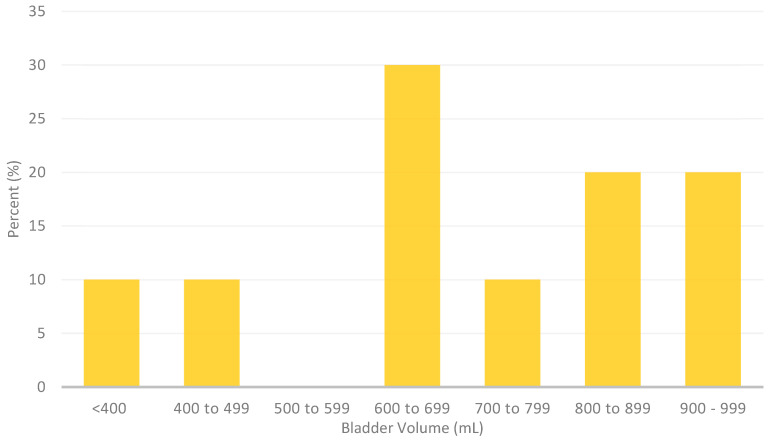
Urine volumes detected on ultrasound bladder scans prior to the first intermittent catheterization post-operatively (*n* = 10).

Post-operatively, of the 16 patients who experienced POUR, only 10 had a documented diagnosis with an ultrasound scan. The detected bladder volumes varied from 298 mL to 999 mL, with a mean volume of 700 mL and a median volume of 695 mL; 20% of these patients were detected with <500 mL on ultrasound, 40% were detected with 600 to 800 mL, and another 40% were detected with >800 mL (Figure 1). The remaining six patients were catheterized post-operatively without any bladder ultrasound scan being recorded and were decided on a clinical basis.

### 3.4. Complications of Post-Operative Urine Retention

None of the 92 patients in this study developed a UTI or AKI during their admission. There were also no documented cases of deep vein thrombosis (DVT) or prosthetic joint infection (PJI) in either groups of patients.

## 4. Discussion

### 4.1. Key Findings

**Figure 2:**
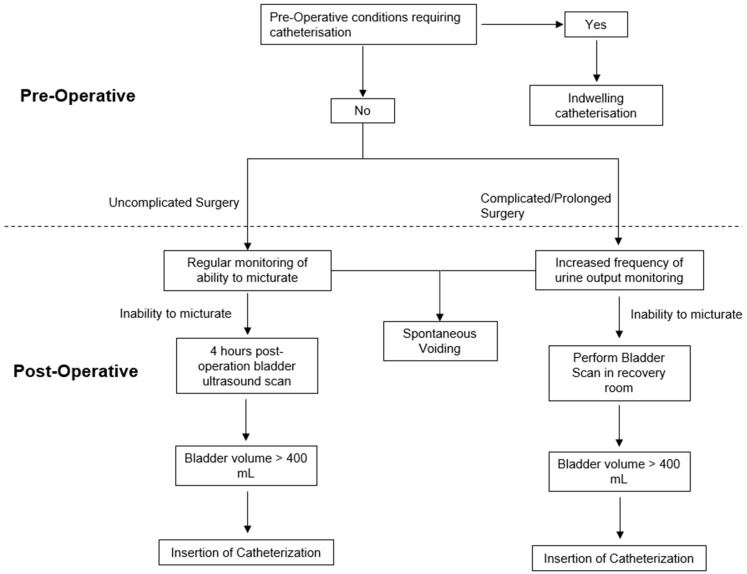
Proposed algorithm for the investigation and management of patients with POUR post-hip or knee arthroplasty using an ultrasound-guided bladder scan.

As previously reported in another study, the incidence of POUR in hip and knee arthroplasty ranges between 10.7 and 77.8% [17]. Our study results corroborate this, with 17.4% developing POUR. The variability of criteria used to diagnose POUR makes it difficult for clinicians to promptly diagnose and manage it. Some studies defined POUR by bladder volumes of 500 mL or more [9], whereas others diagnose POUR clinically with the presence of abdominal discomfort, hypotension, inability to micturate, and a bladder scan qualitatively showing an extended bladder [18]. In our study, patients with POUR had a mean bladder volume of 700 mL. Practices vary among clinicians in our unit. Some follow a protocol of conducting a bladder scan ultrasound 4 h post-operation if patients have not yet micturated, with volumes above 400 mL requiring catheterization. Others prefer to catheterize patients based on clinical decisions, such as abdominal discomfort and the inability to void. However, this approach can leave room for greater interpretation and clinical judgement, potentially resulting in patients with an excessively distended bladder reaching over 999+ mL. POUR can lead to severe hospital complications, such as hypo- or hypertension, cardiac dysrhythmias, UTI, AKI, and long-term myogenic changes to the bladder [19]. However, volumes of less than 1000 mL tend to not cause much harm if diagnosed and treated within 2 h [20]. Additionally, while our study did not demonstrate a significant difference in the length of the inpatient stay between patients who developed POUR and those who did not, other studies have. POUR has been shown to prolong the length of the hospital stay from 6 days to 7 days [9]. This prolongation may reduce hospital bed availability and lower surgical patient turnover. Therefore, it is crucial for clinicians to diagnose POUR promptly or possibly identify patients who are more likely to require more frequent post-operative monitoring. Hence, we propose aiming to conduct an ultrasound scan 4 h post-operation and catheterizing patients who reach the threshold of 400 mL immediately to avoid further bladder distension—see the algorithm in Figure 2.

The anaesthesia team should be attentive to the relationship between post-operative urinary retention (POUR) and the volume of intravenous fluids administered. Research suggests that surpassing an intraoperative fluid volume of 2 L is significantly linked to POUR development, irrespective of other risk factors [21]. Conversely, age-related comorbidities, dehydration, and surgical blood loss heighten the risks associated with perioperative hypovolemia and hemodynamic instability [22]. However, without a comprehensive understanding of the concept of fluid responsiveness, clinicians may inadvertently administer excessive intraoperative fluids, increasing the likelihood of fluid overload and associated complications, such as POUR, or under-resuscitation causing haemodynamic instability. Various methods, including static indices, like the central venous pressure (CVP) and pulmonary artery pressure (PAP), as well as dynamic indices, such as stroke volume variation and pulse pressure variation, can serve as potential predictors for clinicians to assess a patient’s fluid responsiveness [23].

It has also been reported in multiple studies that the duration of surgery is a significant risk factor for the development of POUR, which is consistent with the findings of this study [24]. This could be due to various reasons—more blood loss in a complex surgery requiring more fluid replacement, a higher dose and the accumulation of anaesthetic drugs, or a longer duration of bladder filling [25]. This information could certainly aid clinicians in managing patients post-operatively. Therefore, we propose an increased frequency of urine output monitoring by performing a bladder ultrasound scan in the recovery room for patients who have undergone surgeries lasting longer than 120 min. Furthermore, clinicians may also consider pre-operatively catheterizing patients who are likely to undergo a complicated and prolonged surgery or have a previous history of urine retention post-operation (Figure 2).

In terms of the anaesthetic techniques performed, our results do not show a statistically significant difference between spinal and general anaesthetics concerning the occurrence of POUR (*p* = 0.2). However, this is not consistent with a previous study found online. That study suggests that spinal anaesthesia, such as bupivacaine and tetracaine, may result in a delay in the return of bladder function leading to distension of the bladder and eventually lead to POUR. This results in patients, having undergone spinal anaesthesia, to have a significantly increased risk of POUR compared to general anaesthetics [26]. In contrast, there was another study that found the percentage of urinary retention to be 5.3% in the GA group, 0% in the SA group, and 21.4% in the epidural group [15]. This shows that there are various mixed findings regarding the significance of the anaesthetic technique in relation to POUR. A possible explanation for the mixed findings could be due to the use of a fast-track recovery pathway that is developed by the Enhanced Recover After Surgery (ERAS) Society. This pathway strives to optimize preoperative patient education, the anaesthetic technique, a combination of an opioid-sparing analgesic approach, post-operative care, and early mobilization. It was shown by ERAS that the fast-track recovery pathway has a reduced length of hospital stay from 4–10 days to 1–3 days for patients who had undergone hip/knee replacement [27]. Research indicates that the use of intrathecal opioids as part of regional anaesthesia can impact bladder control and potentially exacerbate urinary retention. Kuipers et al. demonstrated that the administration of intrathecal opioids, such as morphine and sufentanil, can diminish bladder function by suppressing detrusor contractility and reducing the urge to urinate. Therefore, in their ERAS protocol, they opted for GA over SA to minimize the likelihood of POUR. This approach has yielded promising results, with only 5.5% of patients experiencing POUR in their study, compared to a wide range of reported incidence rates online, spanning from 10.7% to 77.8% [28].

The risk of urinary tract infection following bladder catheterization post-arthroplasty is a significant concern, given its status as a well-recognized post-operative complication [29,30]. Hematogenous spread from the urinary tract poses the potential threat of infecting the prosthetic joint or causing systemic dissemination, leading to severe complications, like sepsis [31]. Post-operative bacteriuria has been noted to elevate the risk of prosthetic infection by 3 to 6 times [8]. There are some reports suggesting that opting for indwelling catheterization in patients at a risk of POUR might offer advantages over intermittent catheterization, with a reduced POUR risk and no alteration in UTI incidence [32]. Comparing 24 h indwelling catheterization to intermittent catheterization, there is no discernible difference in the UTI risk [33]. However, intermittent catheterization carries an elevated risk of undiagnosed bladder overdistension, heightening the risk of permanent bladder dysfunction. Although POUR is a common occurrence after THA/TKR, routine pre-operative catheterization is not recommended unless specific high-risk factors are present, such as a prolonged surgery duration [34]. In cases where POUR arises and catheterization becomes necessary, intermittent catheterization may still be preferred as it has been shown to be more cost-effective than indwelling catheterization [33,34].

### 4.2. Limitations of Study

One significant limitation of our study is the small sample size, consisting of only 92 patients. This limited sample size resulted in insufficient statistical power to perform extensive regression analysis, thus hindering our ability to establish definitive predictive relationships between risk factors and outcomes. Moreover, being a single-centre study, our findings are more susceptible to bias, such as selection bias or institutional bias, potentially compromising the accuracy and reliability of our results. Additionally, the retrospective nature of our project posed challenges in terms of data collection and missing information. Woodend Hospital’s transition from paper to electronic patient records further complicated data retrieval, as variables and information had to be sourced from both databases. Despite these challenges, we anticipate that the shift to digital records will ultimately enhance research accessibility and efficiency in the long run. Furthermore, due to time constraints, we were unable to conduct prospective analyses on patients who experienced POUR to assess potential long-term effects on micturition. This limitation underscores the need for future research to explore the extended implications of POUR on patient outcomes.

### 4.3. Conclusions

Evidently, our study has produced results that remain largely consistent with existing studies that have been previously reported. Exploratory logistic regression analysis has shown that the duration of a THA/TKA is associated with the occurrence of POUR. However, we were unable to significantly identify other key risk factors relating to POUR, indicating that it might not be possible to predict POUR in our situation easily. One resolution would be a recommendation for all patients to routinely receive an ultrasound bladder scan 4 h post-operation if they fail to void, to avoid the late detection of POUR. For patients who have undergone prolonged surgery, it might be recommended to increase the frequency of micturition monitoring within the first 4 h post-operation and to conduct bladder scans within the 4 h threshold if needed.

## Figures and Tables

**Table 1:** Patient demographics and clinical characteristic.

		POUR (*n* = 16)	Non-POUR (*n* = 76)	*p*-Value
Sex	Male Female	5 (31.3%) 11 (68.7%)	27 (35.5%) 49 (64.5%)	0.73
Type of Arthroplasty	THA TKA	10 (62.5%) 6 (37.5%)	28 (36.8%) 48 (63.2%)	0.31
Anaesthetic Technique	Spinal General Spinal & General	12 (75%) 2 (12.5%) 2 (12.5%)	68 (89.5%) 6 (7.9%) 2 (2.6%)	0.17
Pre-Operation Morphine	Yes No	2 (12.5%) 14 (87.5%)	9 (11.8%) 67 (88.2%)	0.88
Co-Morbidities	Cardiovascular related	12 (92.3%)	45 (78.6%)	0.34
	Renal related	1 (7.7%)	11 (21.4%)	
Age		73.2 ± 10.8	69.2 ± 10.9	0.18
Body Mass Index (BMI)		31.2 ± 7.0	30.9 ± 5.2	0.72
Duration of Surgery (mins)		105 ± 34	89 ± 19	0.012
Length of Hospital Stay		4.0 ± 3.0	3.1 ± 4.7	0.51
Duration of Catheter (Days)		2.6 ± 1.4		
Past Urological History	Prostatic Disease	1	2	
	Overactive bladder	1	0	
	Urinary Retention	1	1	

**Table 2:** Univariant logistic regression analysis examining the effect of predictors on post-operative urine retention.

	N	OR	95% CI	*p*
Sex (Female as reference)	92	1.21	0.38–3.86	0.74
Type of Arthroplasty (TKA as reference)	92	0.35	0.12–1.07	0.06
Pre-Operation Morphine (No as reference)	92	1.06	0.21–5.5	0.94
Age (Continuous)	92	0.96	0.91–1.02	0.19
Body Mass Index (BMI) (Continuous)	92	0.99	0.90–1.09	0.84
Duration of Surgery (mins) (Continuous)	92	0.97	0.95–1.00	0.018
Length of Hospital Stay (Continuous)	92	0.97	0.88–1.07	0.51

N = number of patients; OR = Odds ratio; CI = Confidence interval.

## Data Availability

All data are included in this manuscript.
